# A Structured Comparison of the Coalition for Health AI Responsible AI Guide and South Korea’s Trustworthy AI Guideline for Health Care AI Assurance: Comparative Framework Analysis

**DOI:** 10.2196/86220

**Published:** 2026-06-11

**Authors:** Trevor Vigeant, Aidan Tam, Qiming Shi, Jeroan Allison, MinJin Kim, Jin-Ah Sim, Dong Ok Won, DongSoo Shin, David M McManus, Jae Jun Lee, Jae Yong Yu, Adrian H Zai

**Affiliations:** 1 Department of Medicine, UMass Chan Medical School, Worcester, MA, United States; 2 The Massachusetts AI Assurance for Healthcare (MAIAH) Lab, UMass Chan Medical School, Worceter, MA, United States; 3 Department of Medicine, The Chinese University of Hong Kong, Hong Kong, China (Hong Kong); 4 Center for Clinical Translational Sciences, UMass Chan Medical School, Worcester, MA, United States; 5 Department of Population and Quantitative Health Sciences, UMass Chan Medical School, Worcester, MA, United States; 6 College of Nursing, University of Cincinnati, Cincinnati, OH, United States; 7 College of Nursing, GFRC, Hallym University, Chuncheon, Republic of Korea; 8 Graduate School of Public Health and Healthcare Management, The Catholic University of Korea, Seoul, Republic of Korea; 9 Department of AI Convergence, Hallym University, Chuncheon, Republic of Korea; 10 Healthcare AI Assurance Lab, Hallym University, 1 Hallymdaehak-gil, Chuncheon, Republic of Korea, 33 248-2035; 11 Hallym University Chuncheon Sacred Heart Hospital, Chuncheon, Republic of Korea; 12 Department of Anesthesiology, Hallym University, Chuncheon, Republic of Korea; 13 Research Institute for Data Science and AI (Artificial Intelligence), Hallym University, Chuncheon, Republic of Korea; 14 Division of Data Science, Hallym University, Chuncheon, Republic of Korea; 15 Department of Emergency Medicine, Hallym University, Chuncheon, Republic of Korea

**Keywords:** artificial intelligence, AI, health policy, clinical decision support, responsible AI, AI assurance

## Abstract

**Background:**

Trustworthy artificial intelligence (AI) in health care requires assurance frameworks that translate ethical principles into measurable governance and evaluation practices. While a growing number of AI assurance frameworks have been proposed, they differ substantially in governance structure, institutional embedding, and implementation mechanisms, reflecting differences in intended purpose and use. To date, few studies have applied standardized, rubric-based evaluation criteria to systematically compare how assurance instruments with different institutional origins operationalize ethical principles across the AI lifecycle.

**Objective:**

This study aimed to develop and apply a structured, rubric-based evaluation instrument to compare 2 health care AI assurance instruments, including the Coalition for Health AI (CHAI) responsible AI guide, a voluntary consortium-based instrument, and South Korea’s Trustworthy AI guideline, a government-issued instrument.

**Methods:**

A 7-dimension evaluation rubric was developed based on a synthesis of established international AI assurance and governance instruments. The rubric covered core principles, AI lifecycle coverage, governance context, stakeholder breadth, operational maturity, instrument design and tools, and public accessibility. Seven independent evaluators with expertise in health care AI governance assessed each instrument using a 5-point ordinal rating scale (1=absent-5=comprehensive). Each evaluator independently scored the materials using a standardized rubric. Discrepancies were resolved through structured consensus discussions, with reference to rubric definitions and source documents. Final scores were determined based on documented evidence, requiring full consensus rather than averaging. Interrater reliability was assessed using Fleiss kappa.

**Results:**

Both instruments demonstrated strong alignment in core principles (CHAI: 4; Trustworthy AI Guideline: 5) and stakeholder breadth (both: 4). The government-issued Trustworthy AI Guideline exhibited broader AI lifecycle coverage (5 vs 4), a more formalized governance context (5 vs 3), and higher operational maturity (4 vs 2), reflecting stepwise oversight and formal embedded oversight mechanisms supported by legislation. In contrast, the voluntary CHAI instrument demonstrated greater emphasis on instrument design and implementation tools (4 vs 3) and higher public accessibility (5 vs 3), driven by open-access resources such as assurance standards guides and applied model cards. Interrater agreement of independent ratings was moderate to substantial (Fleiss kappa=0.47-0.64; *P*<.001), indicating consistent scoring patterns among evaluators.

**Conclusions:**

This comparative analysis indicates that voluntary and government-issued AI assurance instruments operationalize trustworthy AI principles in distinct but complementary ways. Voluntary instruments emphasize flexible tools and accessible implementation resources, while government-issued guidelines embed assurance functions within formal governance and oversight structures. Rather than representing competing models, these approaches address different assurance needs across the AI lifecycle. By identifying concrete areas of alignment and divergence, this study supports a more coherent comparison of assurance practices and highlights potential opportunities for alignment across documentation structures and evaluation approaches that can support safe, equitable, and scalable deployment of health care AI across diverse institutional contexts.

## Introduction

The widespread integration of artificial intelligence (AI) in health care presents a dual reality. On one hand, AI offers new opportunities for decision-making, diagnostic innovation, and therapeutic advancement [1-7]. On the other hand, it introduces significant risks to patient safety, equity, and trust [5,6,8,9]. Recent analyses indicate that US Food and Drug Administration (FDA)–cleared medical AI may experience significant performance degradation when deployed outside their original development settings, underscoring the need for rigorous external validation and ongoing monitoring to ensure safety and effectiveness across diverse clinical environments [10-14]. Similar concerns have been reported in international deployments, where models trained in one context underperform when applied in another, highlighting the global nature of this challenge [15-17]. Realizing the benefits of medical AI depends on effective assurance instruments, which are systematic and evidence-based processes that verify AI systems are safe, fair, transparent, and reliable for both patients and clinicians [18-21]. Without such assurance, failures in AI systems can cause harm, worsen disparities, and undermine confidence in health care delivery. Although the need for trustworthy AI is widely recognized, and several international guidelines have recently emerged, the absence of broadly harmonized and consistently operationalized assurance standards for health care AI remains a significant barrier to the safe, equitable, and trustworthy deployment of medical AI worldwide [21-25].

In recent years, a range of international organizations, consortia, and public authorities have developed frameworks to promote responsible and trustworthy AI in health care. Examples of influential governance references include the World Health Organization (WHO)’s guidance on ethics and governance of AI for health [26], the emerging European Union AI Act [27], International Organization for Standardization / International Electrotechnical Commission (ISO/IEC) standards for AI management systems [28], and Association of Southeast Asian Nations (ASEAN) regional AI policies focused on health care governance [29]. Among these diverse efforts, 2 prominent health care AI assurance frameworks illustrate contrasting governance and implementation models: a voluntary, consortium-developed instrument and a government-issued guideline supported by formal oversight mechanisms. The Coalition for Health AI (CHAI), a public-private consortium, has released a draft responsible AI guide outlining ethical and technical considerations across the AI lifecycle and includes practical tools such as the Assurance Standards Guide and applied model cards [30,31]. These efforts complement other related initiatives, including the FDA’s action plan for AI- and machine learning–based software as a medical device and academic consensus instruments such as fairness, universality, traceability, usability, robustness, and explainability for AI (FUTURE-AI) [24,32]. The Ministry of Science and Information and Communication Technology (ICT) in South Korea has introduced the Trustworthy AI guideline. This guideline functions as a nationally endorsed assurance instrument rather than as a comprehensive regulatory regime for medical AI. This government-led instrument translates ethical principles into operational practices, supported by tools such as the AI Ethics Self-Checklist and the AI Ethics Impact Assessment instrument [33-38]. This guideline is reinforced by recent legislation, including the AI Basic Act, which establishes broader regulatory requirements for AI oversight [37,39].

Although the CHAI responsible AI guide and the Trustworthy AI guideline share core goals such as transparency, fairness, and accountability, they have evolved with limited cross-referencing or shared infrastructure. In this study, the CHAI guide emphasizes voluntary adoption and implementation tools, whereas the Trustworthy AI guideline embeds assurance expectations within a more formal governance and oversight structure [35-37,40].

These differing approaches influence how AI systems are validated and considered for use across distinct regulatory and institutional contexts. As the development and evaluation of clinical AI increasingly occur within specific regulatory or organizational assurance frameworks, variations in governance structures and evaluation practices naturally arise. Understanding how such differences are reflected in existing assurance frameworks is important for contextualizing their scope, assumptions, and applicability, particularly as health care AI is developed and assessed across diverse regulatory environments [41].

Recent instruments such as AI for Implementation and Management of Patient-centered AI and Clinical Translation Systems, Translational Evaluation of Healthcare AI, and multicriteria models have advanced structured approaches for evaluating individual AI systems [42-44]. However, fewer studies have systematically compared how institutionally distinct assurance instruments operationalize trustworthy AI principles across the health care AI lifecycle. In this study, we treat assurance instruments themselves as a distinct unit of analysis for structured evaluation. To address this gap, we apply a structured, rubric-based evaluation to compare the CHAI responsible AI guide and the Trustworthy AI guideline, focusing on how differences in institutional origin and governance context shape assurance expectations, implementation tools, and lifecycle coverage. Our goal is to identify actionable points of alignment and divergence that may support future harmonization of assurance documentation and evaluation approaches across diverse health care settings.

## Methods

### Overview and Comparison Instrument

To operationalize the study aim, we conducted a structured comparative analysis of 2 health care AI assurance instruments with distinct institutional origins: the voluntary, consortium-developed CHAI responsible AI guide and the government-issued Trustworthy AI guideline. Both instruments were analyzed across a predefined set of assurance dimensions to characterize similarities, differences, and opportunities for interoperability and alignment.

### Ethical Considerations

This study consisted of a comparative analysis of publicly available health care AI policy and governance documents and did not involve human participants, personal health information, or identifiable individual-level data. Accordingly, the study did not meet the definition of human subjects research under institutional and federal criteria and did not require institutional review board review or approval. Informed consent, privacy protections, and participant compensation were not applicable.

### Instrument Selection

CHAI and the Trustworthy AI guidelines were selected because they represent mature, health care–specific assurance instruments with contrasting governance and implementation models and publicly available supporting tools. These instruments illustrate 2 distinct approaches to operationalizing trustworthy AI in health care, one consortium-driven and voluntary and the other government-issued and procedurally structured. While other jurisdictions are critical to broader harmonization efforts, this study was designed as an initial comparison focused on 2 institutionally distinct health care AI assurance instruments to enable depth of analysis and methodological clarity. The unit of analysis in this study is the assurance instrument. We examined how 2 health care–relevant instruments with contrasting institutional origins operationalize trustworthy AI principles through governance structure, lifecycle coverage, and implementation tools.

### Instrument Descriptions

We analyzed publicly available documentation from both instruments, including the following materials:

#### CHAI

CHAI provides resources, including the Responsible AI guide, Assurance Standards Guide (2023), applied model card templates, and related online resources. CHAI is a multistakeholder consortium led by academic medical centers, technology companies, and federal agencies, including the National Institutes of Health (NIH) [45]. It provides structured, lifecycle-based assurance resources intended to support responsible development and deployment of clinical AI tools. Key resources include the Assurance Standards Guide and applied model card templates, which outline ethical and technical considerations across AI development and deployment while enhancing transparency and usability in health care settings [30,31]. This lifecycle-based approach supports assurance activities across model development, validation, deployment, and monitoring.

#### Trustworthy AI Guideline

The Trustworthy AI guideline includes the National Guidelines for AI Ethics (2023), the AI Ethics Self-Checklist, and the AI Ethics Impact Assessment instrument. The guideline was developed by the Ministry of Science and ICT in South Korea and exemplified a government-led model of assurance [33]. The instrument integrates ethical principles into structured governance practices and is supported by implementation tools such as the AI Ethics Self-Checklist and the AI Ethics Impact Assessment instrument, which generate standardized evaluation outputs. These tools provide a streamlined and standardized assurance pathway for developers and evaluators.

Documents were retrieved from official websites (Ministry of Science and ICT) and supplemented with publicly available tools and supporting materials [45,46].

### Analytic Approach

Initially, 11 concepts and structural components were extracted from the source materials and coded into predefined comparison dimensions adapted from international assurance frameworks, including the WHO AI Ethics Guidance, the National Institute of Standards and Technology (NIST) AI risk management framework, FUTURE-AI, and ISO/IEC 23894. Concepts that appeared in more than half of these frameworks were retained, resulting in 5 base dimensions.

The selection of source frameworks was based on their relevance to health care AI governance and their recognition as widely used international reference instruments. The threshold of retaining concepts present in more than half of these frameworks was used as a pragmatic criterion to capture consistently emphasized dimensions while minimizing inclusion of framework-specific elements.

The details of this dimension-presence matrix are provided in Multimedia Appendix 1.

### Rubric Development Process

The evaluation rubric was developed in 3 sequential stages.

#### Stage 1 (Initial Dimension Derivation)

Five base dimensions were identified through a documentation-oriented review of international AI assurance and governance frameworks, including WHO AI Ethics Guidance, AI risk management framework, FUTURE-AI, and ISO/IEC 23894. Concepts that appeared in more than half of these instruments were retained to establish an initial 5-dimension rubric.

#### Pilot Phase (Rubric Refinement)

A pilot review was conducted using a limited subset of instrument materials to assess rubric coverage and identify gaps in practical applicability. During pilot application of the preliminary rubric, evaluators identified 2 recurring gaps. First, the draft rubric did not adequately distinguish whether an instrument had translated general principles into repeatable implementation processes or institutionalized workflows. Second, it did not capture whether supporting materials were sufficiently accessible for external review and practical use by stakeholders. These additions ensured that the rubric captured not only the presence of governance principles but also their operationalization and accessibility in practice.

#### Stage 2 (Final Rubric Finalization)

Two additional dimensions—operational maturity and public accessibility—were incorporated to address these gaps. All pilot scores were discarded, and both instruments were fully reevaluated from scratch by all evaluators using the finalized 7-dimension rubric.

The pilot phase consisted solely of an exploratory review conducted to refine rubric structure and definitions and did not contribute to any reported scores. The finalized evaluation rubric consisted of 7 assurance dimensions (Table 1):

1. Core principles: foundational ethical values such as fairness, safety, transparency, and accountability.
2. AI lifecycle coverage: the extent to which the instrument addresses development, validation, deployment, and postdeployment monitoring stages.
3. Instrument design and tools: availability and structure of operational supports such as checklists, model cards, or reporting mechanisms.
4. Governance context: stakeholder composition, governance structures, and regulatory or non-regulatory positioning relevant to the instrument’s intended institutional role.
5. Stakeholder breadth: the range of actors engaged, including developers, clinicians, regulators, patients, and industry.
6. Operational maturity: the extent to which the instrument is translated into established and repeatable operational practices, including defined procedures, implementation workflows, and institutional embedding.
7. Public accessibility: the degree to which the instrument and its supporting materials are publicly available, clearly documented, and usable by external stakeholders without privileged access.

Each instrument was then assessed across the 7 dimensions using a 5-point ordinal scale where 1 indicates absent and 5 indicates comprehensive. This scale was chosen to balance granularity and interpretability, consistent with comparative policy analysis practices. All dimensions were weighted equally to avoid introducing assumptions about the relative importance of specific assurance domains, given the exploratory nature of this study.

**Table 1.** Scoring criteria for assurance dimensions.

Dimension	1=Absent	2=Limited	3=Partial	4=Substantial	5=Comprehensive
Core principles	No explicit principles	One or 2 principles; no detail	Several principles; not operationalized	Broad set; some linked to practice	Broad set; consistently translated into actionable guidance
AI^a^ lifecycle coverage	One lifecycle stage only	>1 stage; narrow scope	Multiple stages, but missing validation or monitoring	Most stages; limited detail available validation/monitoring	Full coverage of all major stages
Instrument design and tools	No tools provided	Tools mentioned; not usable	At least one tool available; limited scope	Multiple tools mapped to lifecycle stages; not standardized	Multiple structured tools are consistently linked to lifecycle stages and available
Governance context	No governance described	references; no roles	Stakeholders named; loose roles	Clear structures; weak enforcement	Clearly defined governance structures with explicit authority, accountability mechanisms, and decision-making processes, whether implemented through regulatory or nonregulatory institutional arrangements
Stakeholder breadth	Single audience	Two groups mentioned; minimal detail	Multiple groups referenced; not systematic	Broad set but missing key groups	Explicit engagement of diverse stakeholders (clinicians, developers, regulators, patients, and industry)
Operational maturity	Conceptual only	Pilots planned; not executed	Piloted in limited settings	Implemented nationally or institutionally; uneven uptake	Established multi-institutional or national use, sustained
Public accessibility	Not publicly available	Only summary information	Some documentation; limited tool access	Most materials are available; some are restricted	Full public access to guides, tools, and platforms

a AI: artificial intelligence.

### Evaluator Characteristics

Seven evaluators (labeled E1-E7 in Table 2), including 4 based in the United States and 3 based in South Korea, with expertise in AI assurance, health informatics, and international regulation, reviewed all study materials and rated each instrument across 7 dimensions using a standardized evaluation form (Table 1) with a 1‐5 point scale [33,45]. All evaluators were provided with the same standardized documentation set and scoring criteria. Evaluators were based in the United States and South Korea and included individuals with expertise in AI assurance, health informatics, and health policy. This multidisciplinary composition was intended to support balanced interpretation of governance, implementation, and institutional context across the 2 instruments.

**Table 2.** Individual evaluator ratings (E1-E7) for Coalition for Health AI (CHAI) responsible AI guide and Trustworthy AI guideline across 7 assurance dimensions. Each evaluator independently scored both instruments on a 1‐5 scale (1=absent-5=comprehensive) based on evidence from publicly available documents.

Instruments and assurance dimensions		E1	E2	E3	E4	E5	E6	E7	Mean (SD)	Agreed score
CHAI^a^ (κ=0.64)										
Core principles		4	4	4	4	4	4	4	4.00 (0.00)	4
AI^b^ lifecycle coverage		4	4	4	3	4	4	3	3.71 (0.49)	4
Instrument design and tools		4	4	5	4	4	5	4	4.29 (0.49)	4
Governance context		3	3	4	3	4	3	3	3.29 (0.49)	3
Stakeholder breadth		4	4	5	4	5	4	4	4.29 (0.49)	4
Operational maturity		3	2	2	2	2	3	2	2.29 (0.49)	2
Public accessibility		5	5	5	5	5	5	5	5.00 (0.00)	5
Trustworthy AI guideline (κ=0.47)										
Core principles		3	3	5	5	5	5	5	4.43 (0.98)	5
AI lifecycle coverage		5	4	5	5	5	5	5	4.86 (0.38)	5
Instrument design and tools		4	3	4	3	3	4	3	3.43 (0.53)	3
Governance context		4	5	5	5	5	5	5	4.86 (0.38)	5
Stakeholder breadth		4	3	3	4	3	4	4	3.57 (0.53)	4
Operational maturity		4	4	4	4	4	4	4	4.00 (0.00)	4
Public accessibility		4	4	3	3	3	3	3	3.29 (0.49)	3

a CHAI: Coalition for Health AI.

b AI: artificial intelligence.

To reduce potential professional or intellectual bias, no evaluator scored an instrument they had authored or directly governed. All scoring was conducted independently prior to any group discussion. Structured consensus discussions required explicit reference to predefined rubric criteria and source documents, and disagreements were resolved based on documented instrument features rather than evaluator preference. Interrater agreement was quantified using Fleiss kappa on preconsensus ratings, after which final agreed scores were reported. However, none of the evaluators participated in scoring an instrument they had authored or governed.

For each assurance dimension, evaluators first recorded their scores independently. If all evaluators assigned identical scores, that value was adopted directly as the final agreed score. When scores differed, a structured consensus discussion was conducted in which evaluators revisited the scoring criteria and source documents to agree on a single ordinal score that best reflected the collective interpretation of the instrument.

### Statistics

To describe the initial distribution of ratings, descriptive statistics with mean (SD) were calculated for each dimension, as shown in Table 2. Mean (SD) values were solely to summarize the distribution of initial independent ratings and were not used to derive or adjust the final consensus scores. In addition, interrater agreement was assessed using Fleiss kappa for multiple raters to quantify consistency across evaluators.

### Outputs

**Figure 1.**
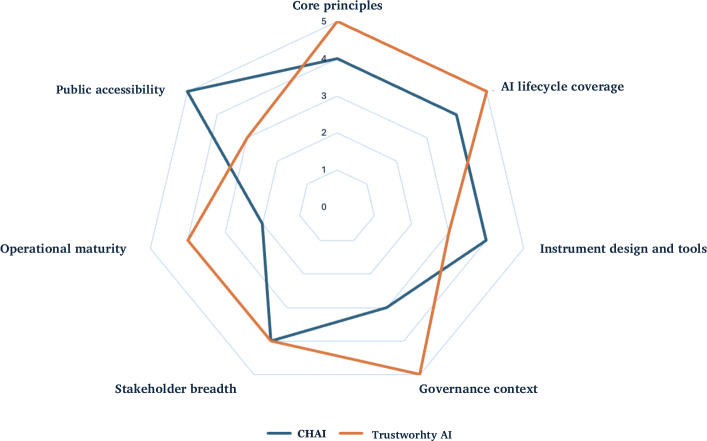
Comparative radar chart of Coalition for Health AI (CHAI) and Trustworthy AI instruments across 7 assurance dimensions. AI: artificial intelligence; CHAI: Coalition for Health AI.

Results were synthesized into two primary outputs: (1) a side-by-side mapping of similarities, instrument-specific features, and actionable opportunities for harmonization as summarized in Table 3, and (2) a radar chart depicting relative strengths across assurance dimensions, providing a visual summary of areas of alignment and divergence as illustrated in Figure 1.

**Table 3.** Consensus summary of comparative scores across 7 assurance dimensions for Coalition for Health AI (CHAI) responsible AI guide and Trustworthy AI guideline.

Assurance dimension	CHAI^a^	Trustworthy AI^b^ guideline	Key similarities / differences	Opportunities for harmonization
Core principles	Score: 4; broad set of principles (transparency, fairness, and accountability) referenced and partially operationalized.	Score: 5; a comprehensive set of principles explicitly defined and translated into guidance.	Both emphasize core ethical values; Trustworthy AI guideline is more explicit and prescriptive.	Trustworthy AI guideline’s explicit codification could strengthen CHAI; CHAI’s integration into tools could enhance Trustworthy AI guideline.
AI lifecycle coverage	Score: 4; emphasizes development and evaluation; limited postdeployment monitoring.	Score: 5; stepwise oversight across all lifecycle stages.	Both cover development and validation; Trustworthy AI guideline extends more fully into deployment and monitoring.	Adapt Trustworthy AI guideline’s lifecycle oversight into CHAI; Trustworthy AI guideline could incorporate CHAI’s flexibility.
Instrument design and tools	Score: 4; provides practical tools (Assurance Standards Guide, model cards).	Score: 3; includes checklists and impact assessments, but higher-level.	Both provide tools; CHAI’s are more technical, Trustworthy AI guidelines more procedural.	Combine CHAI’s technical tools with Trustworthy AI guidelines structured assessments.
Governance context	Score: 3; multistakeholder consortium governance with distributed accountability and structured coordination, without centralized institutional authority or formally codified enforcement mechanisms.	Score: 5; public-sector governance structure with formalized institutional authority, clearly defined accountability pathways, and explicitly assigned implementation responsibilities.	Both engage multiple actors; Trustworthy AI guidelines have regulatory authority, CHAI relies on voluntary adoption.	Combine Trustworthy AI guidelines regulatory clarity with CHAI’s multistakeholder flexibility.
Stakeholder breadth	Score: 4; multiple stakeholders (clinicians, developers, regulators, and industry); limited patient engagement.	Score: 4; broad set, with strong government and professional involvement.	Both engage wide stakeholders; patient/public voices less prominent in both.	Expand stakeholder engagement to systematically include patients and communities.
Operational maturity	Score: 2; draft stage, limited uptake in US systems.	Score: 4; national-level implementation through legislation.	Both instruments still evolving; Trustworthy AI guidelines further advanced in institutionalization.	United States could follow Trustworthy AI guidelines maturity pathway; Trustworthy AI guidelines could adopt CHAI’s iterative piloting practices.
Public accessibility	Score: 5; full open access to guides, tools, and templates.	Score: 3; guidelines public, tools less accessible.	Both make instruments available; CHAI’s materials more open and detailed.	Trustworthy AI guidelines could expand open-access dissemination; CHAI could learn from Trustworthy AI guidelines regulatory integration.

a CHAI: Coalition for Health AI.

b AI: artificial intelligence.

The chart compares the CHAI instrument and Trustworthy AI guidelines based on 7 key assurance dimensions, including core principles, AI lifecycle coverage, instrument design and tools, governance context, stakeholder breadth, operational maturity, and public accessibility. Each dimension is scored on a scale from 1 (lowest) to 5 (highest) based on the extent and depth of coverage indicated in publicly available instrument documents. Shaded areas represent the relative strengths of each instrument, highlighting areas of alignment and relevance to cross-institutional learning.

## Results

### Overall Comparison

The structured analysis revealed both convergence and divergence between the CHAI responsible AI guide and the Trustworthy AI guidelines. Table 3 summarizes similarities, differences, and opportunities for harmonization across the 7 assurance dimensions. Figure 1 presents a radar chart comparing overall scoring patterns between the 2 instruments. Although both instruments are grounded in shared commitments to transparency, fairness, and accountability, they differ substantially in how these principles are operationalized through governance structures, lifecycle coverage, and implementation tools. These findings reflect differences between specific AI assurance instruments rather than evaluations of national AI regulatory regimes or health system performance.

### Comparative Scoring

Overall, both instruments scored highly on core principles (CHAI: 4; Trustworthy AI: 5) and stakeholder breadth (CHAI: 4; Trustworthy AI: 4), indicating substantial alignment in their ethical foundations and recognition of diverse actors.

Clear divergence emerged in other dimensions. AI lifecycle coverage was more comprehensive in Trustworthy AI guidelines (score: 5), which provides stepwise procedural oversight from development to postdeployment monitoring. CHAI’s responsible AI guide (score: 4) emphasizes development and evaluation but does not provide equally detailed mechanisms for postdeployment monitoring.

Instrument design and tools showed the opposite pattern. CHAI (score: 4) provides practical resources, such as the Assurance Standards Guide and applied model card templates that are designed for operational use by developers and health systems. In contrast, the Trustworthy AI guidelines (score: 3) include checklists and assessment instruments, but these remain relatively high-level and less technically detailed.

The governance context demonstrated a separation between the 2 instruments. CHAI (score: 3) is a consortium-driven initiative that relies on voluntary participation and self-regulation. Trustworthy AI guidelines (score: 5) are embedded in a government-led regulatory environment reinforced by the AI basic act.

Two dimensions newly introduced in this analysis, operational maturity and public accessibility, also revealed divergence. Operational maturity was higher for Trustworthy AI guidelines (score: 4) than for CHAI (score: 2). CHAI’s instrument remains in early phases, with a draft guide released but not yet widely implemented across health systems. Public accessibility favored CHAI (score: 5), which provides open access to guides, tools, and model card templates online. The Trustworthy AI guidelines (score: 3) are publicly available in summary form, but supporting tools and implementation resources are less accessible to external stakeholders. Individual evaluator ratings (E1-E7) for each assurance dimension are presented in Table 2, and the consensus summary of comparative scores is shown in Table 3.

The comparative analysis highlights both the magnitude and consistency of differences between the 2 instruments across several key dimensions. The governance context demonstrated a clear separation between the 2 instruments, with agreed scores of 3 for CHAI and 5 for the Trustworthy AI guidelines, reflecting differences in institutional authority and accountability structures. Operational maturity showed a 2-point gap in agreed scores (CHAI: 2; Trustworthy AI: 4), reinforced by the distribution of independent ratings (mean 2.29, SD 0.49 vs mean 4.00, SD 0.00), indicating differences in the extent to which assurance guidance is embedded in repeatable operational practices. Public accessibility also demonstrated a clear divergence, with CHAI achieving a maximum agreed score (5; mean 5.00, SD 0.00), while the Trustworthy AI guidelines scored lower (agreed score 3; mean 3.29, SD 0.49), reflecting differences in public availability and usability of assurance materials. These findings indicate that meaningful differences are present across multiple dimensions rather than being concentrated in a single area.

Interrater agreement was formally assessed using statistical reliability measures, which demonstrated moderate inter-rater agreement, indicating some variability in expert judgment across dimensions (Fleiss κ=0.64 and 0.47). In the CHAI instrument, low variability was observed, with perfect agreement (mean 4.00, SD 0.00) in “Core Principles” and “Public Accessibility.” Conversely, Trustworthy AI guidelines showed greater divergence, particularly in “Core Principles”; despite an agreed score of 5, it yielded the highest variability (mean 4.43, SD=0.98; range 3‐5). This pattern illustrates how agreed scores provide a single reference classification, while mean and dispersion metrics capture underlying differences in expert interpretation.

Scores represent consensus values derived through discussion and agreement among evaluators following independent scoring. Higher scores indicate broader coverage and operational maturity within each dimension.

### Opportunities for Harmonization

The side-by-side mapping in Table 3 highlights complementary strengths that present opportunities for mutual adaptation. For example, CHAI’s practical toolkits (model cards) could be adapted to strengthen the operational usability of the Trustworthy AI guideline’s guidelines, while the Trustworthy AI guideline’s stepwise lifecycle oversight could inform enhancements to CHAI’s guidance on post-deployment monitoring. Similarly, CHAI’s open-access approach to dissemination could be combined with the Trustworthy AI guideline’s strong regulatory anchoring to produce instruments that are both technically usable and legally enforceable.

## Discussion

### Principal Findings

This comparative analysis highlights how voluntary, consortium-driven, and government-issued assurance instruments operationalize trustworthy AI in health care through distinct but complementary pathways. Although both the CHAI responsible AI guide and the Trustworthy AI guideline are grounded in shared ethical commitments to transparency, fairness, and accountability, they differ meaningfully in how these principles are translated into governance structures, lifecycle oversight, and implementation mechanisms. The observed differences across assurance dimensions can be interpreted in light of differences in institutional origin and intended role. CHAI’s strengths in public accessibility, modular design, and implementation tooling as reflected in publicly available documentation align with its positioning as a voluntary, multistakeholder initiative intended to support early adoption, experimentation, and iterative improvement across diverse health care settings. In contrast, the Trustworthy AI guideline demonstrates stronger documented institutionalization and implementation readiness in governance context, lifecycle coverage, and operational maturity, based on features described in publicly available materials, reflecting their development within a government-led structure supported by formal oversight mechanisms. Taken together, these findings underscore that assurance quality can emerge through multiple institutional pathways rather than through a single optimal governance model.

While the 2 instruments examined in this study are inherently asymmetric in their institutional origins (voluntary consortium vs government-issued), this asymmetry is a deliberate feature of the study design rather than a limitation. The objective is not to compare equivalent regulatory regimes but to examine how similar ethical principles are operationalized under distinct governance models. By selecting instruments that represent different points along the governance spectrum, this comparison enables identification of how institutional context shapes implementation tools, lifecycle oversight, and accountability structures. A comparison restricted to institutionally similar instruments would likely obscure these differences.

To preserve conceptual comparability, this study focuses on assurance instruments rather than broader regulatory or policy frameworks. Here, assurance instruments are defined as structured, operational artifacts (eg, rubrics, checklists, or evaluation frameworks) that can be directly applied to assess AI systems and produce standardized, comparable outputs. In contrast, government-led efforts such as those from the FDA, the National Institute of Standards and Technology, or the WHO primarily articulate regulatory expectations and risk management principles. While these frameworks may inform practice, they do not provide self-contained, standardized evaluation instruments that can be directly applied and scored without additional institutional translation. Including them would therefore introduce heterogeneity in the unit of analysis and compromise the internal consistency of rubric-based comparisons.

Importantly, this comparison is not intended to assess regulatory superiority. Rather, it examines how assurance instruments structure expectations and practices under different governance contexts. Although the Trustworthy AI guideline is government-issued, it functions as a government-endorsed assurance instrument rather than as a comprehensive regulatory regime comparable to medical device regulation. Similarly, CHAI’s voluntary nature does not imply weaker assurance, but instead reflects an alternative governance approach grounded in transparency, professional norms, consensus practices, and practical tooling. Recognizing these distinctions is essential for interpreting differences in governance context and operational maturity scores.

Beyond conceptual alignment, practical harmonization can be achieved through partial interoperability of assurance artifacts. For example, specific elements of CHAI’s applied model card map functionally to components of the Trustworthy AI guideline’s Ethics Impact Assessment:

- Intended clinical use → purpose specification
- Training data description → risk identification and bias review
- Performance stratification and subgroup analysis → fairness evaluation
- Monitoring plan → postdeployment management and oversight

These correspondences illustrate how documentation developed under a voluntary instrument could be reused or adapted within a government-led assurance process, reducing redundant documentation while preserving institutional specificity. More broadly, these artifact-level mappings point to a pragmatic pathway for alignment at the level of documentation and evaluation workflows, without requiring uniform governance structures or regulatory convergence.

Several limitations should be acknowledged. This study examined 2 health care AI assurance instruments selected for their maturity, accessibility, and relevance to health care, and the findings should not be interpreted as a comprehensive assessment of the global trustworthy AI governance landscape. The exclusion of high-authority regulatory and multilateral initiatives may limit the generalizability of these findings and may underrepresent governance models with formal regulatory authority and broader international adoption. In particular, this study did not include several high-authority international or multilateral initiatives, including WHO guidance, FDA-related instruments, and EU (European Union) AI Act–aligned approaches. As such, the results may not generalize to assurance instruments with different scopes, governance structures, or intended uses. In addition, the evaluation reflects expert interpretation of documented assurance features and does not constitute empirical validation of the extent to which stated principles are realized in practice. Health care AI is not a homogeneous category. Large language models, diagnostic classifiers, imaging systems, and clinical decision-support tools may differ substantially in failure modes, monitoring needs, and assurance requirements. For example, large language models raise concerns related to hallucination and persuasive but incorrect outputs, whereas diagnostic classifiers and image-based models may be more strongly affected by shortcut learning, dataset shift, and spurious correlations. The present rubric was designed to compare cross-cutting governance-oriented assurance features rather than modality-specific technical risk controls, which may limit its sensitivity to modality-specific risks. Future work should assess whether the rubric requires adaptation for different AI modalities and deployment contexts. Interpretation of policy and governance documents necessarily involved expert judgment, which introduces subjectivity despite the use of predefined scoring criteria, independent evaluations, and structured consensus procedures. In addition, both instruments are evolving, and the results should be understood as a temporal snapshot rather than a definitive or static assessment. Finally, this study did not evaluate real-world effectiveness, implementation efficiency, or downstream clinical safety outcomes associated with either instrument.

Future work should extend this comparison to additional high-authority and multilateral instruments to better capture the diversity of global governance approaches and assess the generalizability of the findings and incorporate additional independent evaluators, including those with no prior involvement in AI assurance initiatives, to further assess reproducibility and generalizability.

### Conclusion

This study shows that voluntary, consortium-driven, and government-issued approaches to clinical AI assurance operationalize trustworthy AI principles in distinct yet complementary ways. By systematically comparing a tool-focused, nonregulatory assurance instrument with a procedurally embedded, oversight-oriented instrument, we identify practical differences in how assurance expectations are structured, supported, and evaluated. These findings underscore that robust AI assurance can be achieved through multiple institutional pathways rather than a single governance model.

The 7D rubric introduced in this study provides a transparent and reproducible method for assessing AI assurance frameworks and associated instruments, offering a structured basis for comparative analysis across heterogeneous institutional contexts. Together, these contributions support more coherent interpretation and comparison of assurance practices and highlight potential opportunities for aligning documentation and evaluation workflows while respecting differences in governance authority and implementation models in service of safe and equitable deployment of health care AI.

## Supplementary material

10.2196/86220 Multimedia Appendix 1 Coverage of key components from international assurance frameworks.

## Abbreviations

AI: artificial intelligence
ASEAN: Association of Southeast Asian Nations
CHAI: Coalition for Health AI
EU: European Union
EU: European Union
FDA: Food and Drug Administration
FUTURE-AI: fairness, universality, traceability, usability, robustness, and explainability for AI
ICT: Information and Communications Technology
IEC: International Electrotechnical Commission
ISO: International Organization for Standardization
NIH: National Institutes of Health
NIST: National Institute of Standards and Technology
WHO: World Health Organization
