# Supplementary material for: A Structured Comparison of the Coalition for Health AI Responsible AI Guide and South Korea’s Trustworthy AI Guideline for Health Care AI Assurance: Comparative Framework Analysis
Source: JMIR AI. 2026 Jun 11;5:e86220. doi: 10.2196/86220 (PMC13256487; doi:10.2196/86220)
Supplement: Multimedia Appendix 1 [file ai-v5-e86220-s001.docx]

Table S1. Coverage of key components from international assurance frameworks.

|  | **Core Principles** | **AI Lifecycle** | **Design & Tools** | **Governance** | **Stakeholders** | Technical Implantation | Cost-Benefit | Legal Compliance | Cultural Adaptation | Performance Bench. | Risk Protocols |
| --- | --- | --- | --- | --- | --- | --- | --- | --- | --- | --- | --- |
| WHO AI Ethics (2021) | **O** | **O** | **X** | **O** | **O** | X | O | X | O | X | X |
| FDA AI/ML Guidance (2021) | **O** | **O** | **O** | **O** | **O** | O | X | O | X | O | O |
| EU AI Act (2024) | **O** | **O** | **O** | **O** | **O** | X | O | O | X | O | X |
| ISO/IEC 23894 (2023) | **O** | **O** | **O** | **X** | **X** | O | X | X | X | X | X |
| NIST AI RMF (2023) | **O** | **O** | **O** | **O** | **O** | X | X | X | X | O | O |
| IEEE Standards 2857 (2021) | **O** | **O** | **O** | **X** | **O** | X | X | X | X | X | X |
| FUTURE-AI (2025) | **O** | **O** | **O** | **X** | **O** | X | O | X | X | X | X |
| **ACM Code of Ethics (2018)** | **O** | **X** | **X** | **X** | **O** | X | X | X | X | X | X |
| Coverage | **100%** | **87.5%** | **75%** | **50%** | **87.5%** | 25% | 37.5% | 25% | 12.5% | 37.5% | 25% |
